# What Happens After Assessment?

**DOI:** 10.1177/14550725261502834

**Published:** 2026-10-31

**Authors:** Katja Kuusisto

**Affiliations:** 1 Faculty of Social Sciences, Tampere University, Tampere, Finland

The debate article by Björk (2026) asks whether the available evidence warrants the strong recommendation to use the Addiction Severity Index (ASI) in Swedish social services. As I understand it, the concern is not simply whether the ASI can measure the domains it was designed to measure. It is whether evidence about its technical quality and usability is sufficient to justify a recommendation about its use in everyday assessment of support needs. Questions about the evidence supporting different uses of the ASI are longstanding. Mäkelä (2004) questioned whether its psychometric performance supported its proposed clinical, administrative, and research uses. I find this a timely and relevant question also in Finland. The Finnish Current Care Guideline on drug problems lists EuropASI, the European version of ASI, as one possible instrument for systematically assessing drug use and related problems (Huumeongelmat, 2022). This does not establish how widely the instrument is used or what follows from its use in practice. My commentary addresses two questions: *What happens to the information produced during assessment*, and second, *whose understanding of a meaningful outcome guides its evaluation*.

An assessment instrument may help a professional identify concerns that could otherwise remain unspoken. It may also give a client and a professional a shared structure for discussing a complex situation. These are potentially valuable contributions. Yet recognizing a need, deciding what support to offer and ensuring that support is available are different steps. If an instrument is recommended because it is expected to improve support, we need to know more about the connections between these steps. Does the assessment bring important needs into view? Can the client question or add to the account it produces? Does it inform the professional's decisions and subsequent work? Hänninen (2004) approached ASI as a practice of documentation that also shapes how clients’ situations can be described. This perspective raises the question of which aspects of a client's situation the instrument's categories make visible and which may remain outside the assessment.

Our research in Finland on client segmentation tool *Suuntima* (Navigator; currently Omasuuntima; DigiFinland, n.d.) has made me cautious about assuming that information produced in an assessment will necessarily lead to more appropriate services. Developed initially for health care, Suuntima is a digital tool used during an appointment to group clients according to their anticipated support needs. Clients answer questions about their resources and ability to manage in everyday life, while professionals assess their clients’ health status and the complexity of care. Based on these two perspectives, the tool proposes one of four client groups linked to suggested service pathways and forms of support. The resulting group is intended to inform planning with the client (Riihimies et al., 2020, 2023).

Suuntima is neither an adaptation of the ASI nor an alternative to it. The two instruments have different purposes. The reason for bringing Suuntima into this discussion is that its use in services for people with problematic substance use allows us to examine the passage from an assessment to a proposed way of organizing support.

In the SEGMENT project, substance use service professionals reported that Suuntima helped them form a broader picture of a client's situation and supported discussion and cooperation. But they also questioned its suitability for segmenting clients with problematic substance use into groups and considered its implementation inadequate. In the published analysis, all 23 clients assessed with Suuntima were assigned to the *self-acting* group. Examination of their circumstances raised doubts about whether this group adequately represented their service needs, as these clients had severe substance use problems alongside substantial psychological and somatic symptoms. The small sample and limited background information call for care in interpreting that finding (Kuusisto, 2024; Plaami et al., 2025).

These observations do not establish whether Suuntima improves client outcomes or not. The project could not carry out its planned comparison of service pathways as intended. They do, however, show why the fit between an instrument's categories and the circumstances of the people assessed must be examined in the setting where it is used. An instrument may help to open a conversation while its proposed classification remains less helpful for deciding what support is needed.

A further perspective came from our interviews with the managers of substance use services. They saw potential in client segmentation for recognizing different levels of need. However, they questioned whether an initial classification could capture needs emerging over several meetings and whether support would respond to later changes in a client's situation. Most had not used Suuntima themselves in client appointments, and so these findings describe managers’ expectations and concerns rather than observed effects of the tool (Plaami et al., in press).

This distinction between identifying a need and responding to it is central to the debate about assessment instruments. Even when a professional recognizes a client's needs accurately, suitable services may be unavailable. Stenius and Room (2004) linked ASI's adoption in European treatment systems to several aims, including cost-efficiency, scientific legitimacy, professionalization, and system coordination. They described difficulties in turning collected data into information used for service management and research. Their analysis invites a distinction between an instrument's value to a service system and its contribution to meeting a client's needs. A recommendation should therefore make explicit whose purposes the instrument is expected to serve and what evidence supports each intended use.

Research should follow what happens after assessment: How clients and professionals interpret the information, whether it informs service plans and decisions, what support clients actually receive, and whether the assessment is revisited as their situations change.

My second question concerns what we count as a successful outcome of using an assessment instrument, and who participates in defining it. Work on alcohol brief interventions by Shorter et al. (2021) illustrates how different stakeholders can contribute to selecting research outcomes. For assessment in social services, these choices need separate justification, that is, which changes should assessment help bring about, for whom, and through what connection to subsequent support?

For clients of social services, relevant aspects of success may include being heard in an assessment, receiving support corresponding to their needs, and being able to continue with that support as their situation changes. Changes in substance use may also be important, but they do not exhaust the aims of social work. Some outcomes can be measured across clients and services, while others require attention to what a particular client sought help for and whether that need was met.

I welcome the debate article's call for a more transparent relationship between evidence and recommendations. To me, transparency also requires stating which links between assessment and support have been studied and which are still expected rather than demonstrated. An instrument can be useful in a conversation without its effects on later decisions being known. Conversely, routine completion of an instrument cannot by itself show that clients receive the help they need. The strength of a recommendation should reflect both what is known about an instrument and how clearly its expected contribution to practice can be explained.
